# Optimization of binding affinities in chemical space with generative pre-trained transformer and deep reinforcement learning

**DOI:** 10.12688/f1000research.130936.2

**Published:** 2024-02-20

**Authors:** Xiaopeng Xu, Juexiao Zhou, Chen Zhu, Qing Zhan, Zhongxiao Li, Ruochi Zhang, Yu Wang, Xingyu Liao, Xin Gao

**Affiliations:** 1 Computational Bioscience Research Center (CBRC), King Abdullah University of Science and Technology (KAUST), Thuwal, 23955-6900, Saudi Arabia; 2 Computer, Electrical and Mathematical Sciences and Engineering (CEMSE), King Abdullah University of Science and Technology (KAUST), Thuwal, 23955-6900, Saudi Arabia; 3 KAUST Catalysis Center (KCC), King Abdullah University of Science and Technology (KAUST), Thuwal, 23955-6900, Saudi Arabia; 4 Syneron Technology, Guangzhou, China

**Keywords:** Drug design, transformers, reinforcement learning, molecular docking, hit discovery

## Abstract

**Background:**

The key challenge in drug discovery is to discover novel compounds with desirable properties. Among the properties, binding affinity to a target is one of the prerequisites and usually evaluated by molecular docking or quantitative structure activity relationship (QSAR) models.

**Methods:**

In this study, we developed SGPT-RL, which uses a generative pre-trained transformer (GPT) as the policy network of the reinforcement learning (RL) agent to optimize the binding affinity to a target. SGPT-RL was evaluated on the Moses distribution learning benchmark and two goal-directed generation tasks, with Dopamine Receptor D2 (DRD2) and Angiotensin-Converting Enzyme 2 (ACE2) as the targets. Both QSAR model and molecular docking were implemented as the optimization goals in the tasks. The popular Reinvent method was used as the baseline for comparison.

**Results:**

The results on the Moses benchmark showed that SGPT-RL learned good property distributions and generated molecules with high validity and novelty. On the two goal-directed generation tasks, both SGPT-RL and Reinvent were able to generate valid molecules with improved target scores. The SGPT-RL method achieved better results than Reinvent on the ACE2 task, where molecular docking was used as the optimization goal. Further analysis shows that SGPT-RL learned conserved scaffold patterns during exploration.

**Conclusions:**

The superior performance of SGPT-RL in the ACE2 task indicates that it can be applied to the virtual screening process where molecular docking is widely used as the criteria. Besides, the scaffold patterns learned by SGPT-RL during the exploration process can assist chemists to better design and discover novel lead candidates.

## Introduction

The key challenge in drug discovery is to discover new molecules with desirable properties. ^1^ In traditional drug discovery campaigns, high-throughput virtual screening, biochemical assays, physicochemical assays, and *in vitro* profiling of absorption, distribution, metabolism, and excretion (ADME) properties of chemicals are usually conducted. ^2^ However, the chemical space of possible molecules is enormous, with 10 ^23^ to 10 ^60^ potential drug-like molecules and the number of synthesized molecules in the order of 10 ^8^. ^3^ It is infeasible to screen all the molecules to select the desirable ones. Many machine learning tools to predict molecular properties, including binding affinity, drug-likeness, synthetic accessibility, and ADME properties have been integrated into the screening pipelines as key components, ^4^ as they are much faster than traditional computational methods and yield rapid and accurate property predictions. ^3^^,^^5^ Employing these tools has improved the efficiency to virtually screen the chemical libraries, which are generated from available chemical reagents. ^6^^,^^7^ However, the search is still limited to molecules in the chemical libraries.

In recent years, de novo molecular design, especially deep generative models, has witnessed a rapid progress, which can efficiently explore the chemical space and optimize the molecular generation towards desired properties. ^3^^,^^8^^–^^10^ A pioneer work was published in 2018, which employed variational autoencoder (VAE) to learn a continuous representation of the chemical space and used gradient-based optimization to search for functional molecules. ^11^ After that, many methods were developed and the most representative classes include recurrent neural networks, autoencoders, generative adversarial networks, and reinforcement learning (RL). ^3^^,^^4^ Among them, RL methods were shown to be able to optimize the generation of molecules towards desirable properties, including target activity, drug-likeness, molecular weight, synthetic accessibility (SA), and similarity to given molecules. ^4^^,^^6^^,^^12^^,^^13^

Transformer ^14^ is a prominent deep learning method that was first proposed for natural language translation and has made tremendous impact in many fields, such as language modeling, speech processing, and computer vision. ^15^ A decoder-only variant of the transformer, Generative Pretrained Transformer (GPT), stands out among the many transformer variants. It was trained on a large corpus of unlabeled text and able to generate news articles difficult for human evaluators to differentiate from human-written ones. ^16^^,^^17^ Besides, a GPT model fine-tuned with reinforcement learning showed better generative results, with reduced toxic outputs and better truthfulness. ^18^

Several transformer-based methods have been proposed for molecular generation tasks. ^4^^,^^19^^–^^21^ A study formulated the protein-specific molecular generation as a machine translation problem and used amino acid sequences as inputs and simplified molecular input line entry system (SMILES) representation of molecules as outputs. ^19^ The model was pretrained on amino acid sequences of targets and the corresponding SMILES of the binding molecules, and able to generate valid molecules with structural novelty and plausible drug-likeness. Another work also formulated molecular generation as a translation problem, but their goal is to optimize the generation of molecules towards desirable properties. ^21^ They added a desirable property together with the starting molecules as the input and the modified molecules fulfilling the desirable property as the output to train their model. Their results showed that transformers can generate molecules with desirable properties through modifications that are intuitive to chemists. A decoder-only transformer model, MolGPT, was also proposed for molecular generation. ^20^ It was trained on molecules with property conditions and able to generate novel molecules fulfilling the corresponding properties. Another work also used a decoder-only transformer model but targeting multiple properties. ^4^ After pretraining a transformer model, a gated recurrent unit (GRU) model was used to distill it and initiate an RL agent. This agent was then trained to optimize multiple properties through the Reinvent approach. ^13^ The agent can generate novel molecules satisfying multiple property constraints. In summary, these studies showed the advantages of transformers on molecular generation, especially for constrained generation tasks. ^4^^,^^12^

Activity of a compound is the primary consideration for drug discovery, which is induced by binding affinity of a compound to a target. Three approaches are used to estimate binding affinity, including bioassays, quantitative structure activity relationship (QSAR) models and molecular docking. ^22^ *In vitro* bioassays are reliable but often scarce, and QSAR models and molecular docking are usually used for in silico screening process. ^22^ Because transformers are so good at sequence generation and RL has an advantage on optimization tasks, an intuitive idea is to combine transformer and RL to optimize the binding affinity. However, as far as we know, no such studies have been conducted. Two main obstacles may stop researchers from conducting such studies. First, high-end GPUs with large memories are required to conduct such studies. During the RL process, a transformer decoder has to be used to generate a batch of molecules, however, such generation is very memory expensive. Besides, conducting such studies requires interdisciplinary knowledge, including computational chemistry and machine learning expertise. For example, molecular docking is usually used for virtual screening, but is not easy for machine learning experts to perform and interpret; while transformer and RL are widely used in deep learning society, but are hard for computational chemists to grasp and implement.

**Figure 1.**
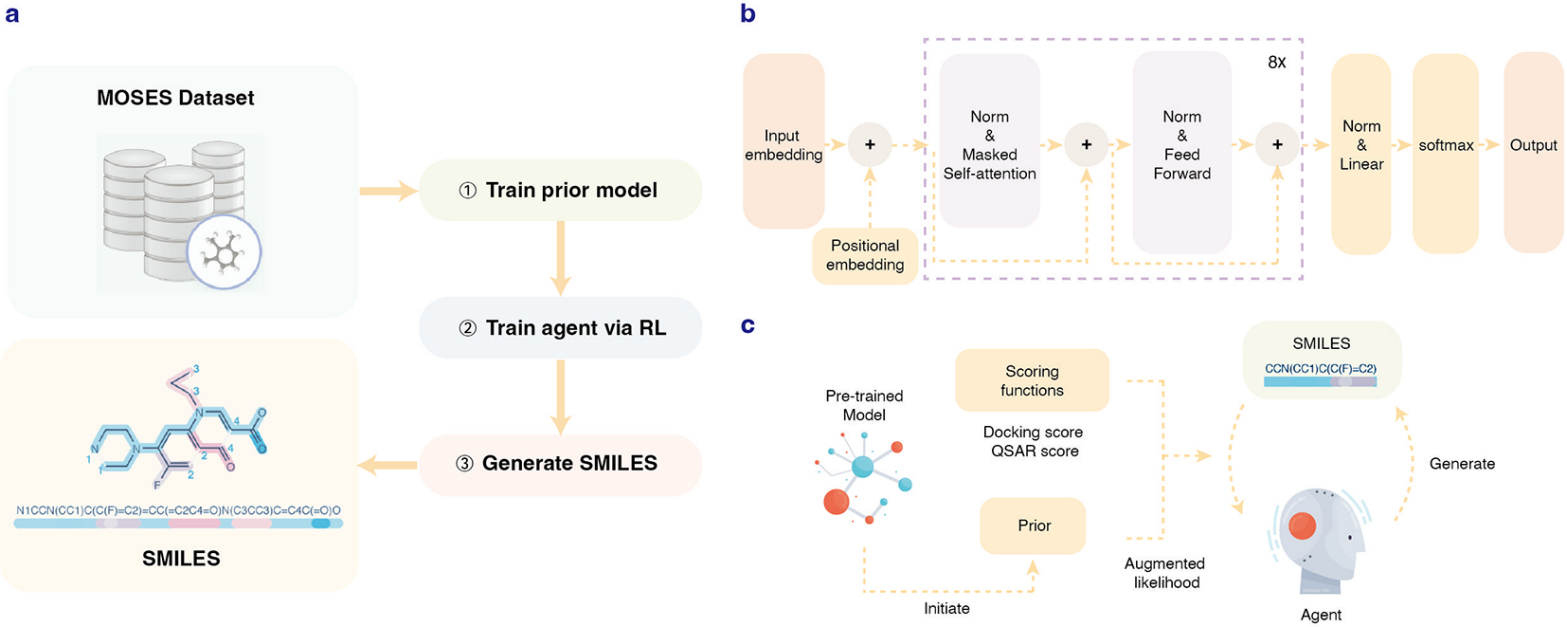
The workflow of SGPT-RL. a) The main workflow. Simplified molecular input line entry system (SMILES) from the Moses benchmark was used to train a prior model. An agent model was then initiated from the prior and trained in a reinforcement learning (RL) fashion to generate molecules with desirable properties. b) The architecture of the prior model. The agent shares the same architecture. c) The pipeline of the RL approach. The prior model was used to initiate the agent model. During each RL step, the agent model was used to generate a batch of SMILES sequences. The generated sequences were evaluated by the prior model and a scoring function to calculate augmented likelihoods, which serve as the feedback to update the agent. In the Dopamine Receptor D2 (DRD2) task, a quantitative structure activity relationship (QSAR) model was used as the scoring function; in the Angiotensin-Converting Enzyme 2 (ACE2) task, ACE2 docking score was used as the scoring function.

In this study, we proposed the first method that combines GPT and RL for molecular generation. We developed a tool named SGPT-RL, which uses a transformer decoder as the policy network of RL agents. The workflow is shown in Figure 1. First, GPT was trained on lead-like molecules to obtain a prior model that learns the chemical space. This prior model was used to initiate the agent, which shared the same decoder model as the policy network. Then, the agent was trained in an RL fashion to optimize the generation of molecules towards desirable properties, as shown in Figure 1c. The agent was used to generate a batch of molecules; the molecules were scored by scoring functions to obtain the target scores; the scores were combined with the prior likelihoods to calculate the losses; the losses that contain both the target score and prior likelihood information were used to serve as the feedback to the agent. During training, the likelihood of the agent to generate molecules with good target scores is increased and those with poor scores decreased. We evaluated SGPT-RL on the Moses distribution learning benchmark and two goal-directed generation tasks. Results on the Moses benchmark showed that the SGPT-RL prior model was able to learn good property distributions and generate molecules with high novelty. The two goal-directed generation tasks are a Dopamine Receptor D2 (DRD2) task, with QSAR model-based activity as the scoring function, and an Angiotensin-Converting Enzyme 2 (ACE2) task, with molecular docking affinity as the target score. In both tasks, the SGPT-RL agents were able to generate valid molecules with high target activities. In the DRD2 task, the SGPT-RL agent was able to explore more scaffolds than the popular Reinvent method; in the ACE2 task, the SGPT-RL agent generated molecules with significantly better docking scores than Reinvent. Besides, we found that the Reinvent agents could not learn effectively after around 100 steps, while the SGPT-RL agents were continuous learning and generating molecules with more ring structures. In addition, we found that the SGPT-RL agents were able to learn some generative patterns, while the Reinvent agents were exploring with strong randomness and no clear patterns could be observed.

## Methods

### Datasets

The dataset to train the prior models was obtained from the Moses benchmark. ^23^^,^^42^ This dataset contains 1.9 million lead-like molecules from the Zinc database. ^24^ The train and test dataset in the Moses benchmark were used for training and testing, which contain 1,584,664 and 176,075 molecules respectively.

Known active molecules that bind with DRD2 or ACE2 were obtained from ExCAPE-DB. ^25^^,^^42^ The 8,036 unique molecules that are known to be active against DRD2 were obtained and 56 unique molecules that are active against ACE2 were retrieved. For these two sets of known active molecules, none of them were found in the Moses training dataset.

### Model architecture

A brief overview of the framework is illustrated in Figure 1a. A transformer decoder prior model was trained on the Moses dataset. This pretrained prior model was used to initiate the agent. During the RL process, the agent model was used to generate molecules, which were scored by the prior network and a scoring function to provide feedback to update the agent. The agent model trained after the final step was used to generate molecules for property distribution analysis.

*The prior network*

In SGPT-RL, a generative pre-trained transformer (GPT) ^26^ was used as the prior model to learn the chemical space. Tokenized SMILES sequences were used to train the model on a next token prediction task.

The GPT model we used is a simplified version of GPT-2, with only ∼6M parameters. The architecture of the model is illustrated in Figure 1b. The model is composed of eight decoder blocks, input and positional embedding before the blocks, a linear layer after the blocks, and a softmax layer before output. Each of the blocks contains a masked multi-head self-attention layer and a fully connected feedforward layer, with residual connections in each of the layers. Layer normalization is conducted in the two layers to normalize the inputs. An embedding size of 256 was used in all layers.

The core of the GPT model is the masked multi-head self-attention layer. In this layer, eight scaled dot-product attention functions facilitate the model to capture key information in a sequence. In the attention function, a query vector Q is used to calculate a dot product with the key vector K and then divided by the key vector length $d_{k}$ . The resulting product value is passed into a softmax function to get the attention weights, which is dot-producted with a value vector V to get the final attention. The formula is shown in Equation 1. ^14^

$$\text{Attention}\left(Q,K,V\right)=\text{softmax}\left(\frac{QK^{T}}{\sqrt{d_{k}}}\right)V$$ (1)

The prior model was trained for ten epochs on the training dataset and evaluated on the testing dataset after each epoch. Cross-entropy loss was used with the AdamW optimizer ^27^ to update the model, with a learning rate of 0.001. A batch size of 1,024 was used to train the model. To generate the SMILES string of a molecule, a start token was fed to the model to predict the next. The generated token was concatenated with previous tokens to predict the next, until an end token was predicted or a maximum sequence length of 140 was reached.

*Training the agent*

The process to generate molecules with desirable properties was framed as a RL problem, and the Reinvent approach was utilized, with the process described below. ^12^ In the RL formulation, the state is the current sequence generated, the action is the next token to add, and the reward is a augmented likelihood calculated from prior likelihood and property scores. The GPT model described in the previous Subsection was used for the prior and the agent, and customized scoring functions for the target properties were used in each of the two tasks.

The loss function to update the agent model is defined as in Equations 2– 3. First, a SMILES sequence *A* was sampled from the agent model with its log-likelihood $\log p{\left(A\right)}_{\text{agent}}$ . Then the SMILES sequence was passed to the prior model to calculate a prior log-likelihood $\log p{\left(A\right)}_{\text{prior}}$ , and evaluated with scoring functions of desirable properties to get a score $S\left(A\right)$ . The score was added to the prior log-likelihood with a coefficient σ to get an augmented log-likelihood $\log p{\left(A\right)}_{\mathrm{aug}}$ , as shown in Equation 2. The idea behind this equation is that the prior log-likelihood is added to preserve the rules learnt from SMILES sequences of molecules, and the score of desirable properties was used to bias the model to generate SMILES of desirable properties.

$$\log p{\left(A\right)}_{\mathrm{aug}}=\log p{\left(A\right)}_{\text{prior}}+\sigma S\left(A\right)$$ (2)

Finally, the squared error between the augmented log-likelihood and agent log-likelihood was used as the loss to update the agent model, as shown in Equation 3.

$$\text{Loss}={\left[\log p{\left(A\right)}_{\mathrm{aug}}-\log p{\left(A\right)}_{\text{agent}}\right]}^{2}$$ (3)

### Evaluation metrics

Five metrics from the Moses benchmark were used to evaluate the models, including validity, uniqueness, novelty, similarity to a nearest neighbor (SNN) and internal diversity (intDiv). The definitions of the metrics are described below. The generated SMILES sequences to be evaluated are denoted by *G*, the training dataset is denoted by *T*, and *n* is the total number of the generated sequences.

• Validity: the fraction of the valid sequences among 10,000 generated sequences.
• Uniqueness: the fraction of the unique sequences among 10,000 valid generated sequences.
• Novelty: the fraction of the unique sequences in G, but not in T.
• Similarity to a nearest neighbor (SNN): evaluates the similarity of the generated molecules to the training molecules. It is the Tanimoto similarity $T\left(m_{G},m_{T}\right)$ between fingerprints of a molecule $m_{G}$ from the generated set *G* and its nearest neighbor molecule $m_{T}$ in the training dataset.

$$\mathrm{SNN}\left(G,T\right)=\frac{1}{n}\sum_{m_{G}\in G}\max_{m_{T}\in T}T\left(m_{G},m_{T}\right)$$ (4)

• Internal diversity (intDiv): assesses the diversity within *G.* It is defined as one minus the averaged Tanimoto similarity of any pair of molecules $m_{1}$ *,* $m_{2}$ in the generated sequences *G.*

$$\text{IntDiv}\left(G\right)=1-\frac{1}{n^{2}}\sum_{m_{1},m_{2}\in G}T\left(m_{1},m_{2}\right)$$ (5)

### Evaluated molecular properties

In our experiments, seven molecular properties were calculated to evaluate the property distributions and used as the optimization goals. All these properties were used to compare the property distributions of molecules. DRD2 activity and ACE2 docking score were used as the scoring functions of the DRD2 and ACE2 tasks, respectively.

DRD2 activity was evaluated with a QSAR model. ^12^ This model is a support vector machine (SVM) classifier with a Gaussian kernel trained on active and inactive molecules. In the modeling, a SMILES is converted into molecules to obtain the Morgan fingerprints using RDKit 2017.09.1. ^28^ The fingerprints were used as the features to build the SVM classifier. It predicts a probability score range from zero to one, with the closer to one the higher DRD2 activity.

ACE2 affinity was calculated using molecular docking as described in Subsection “ *Task 2: structure-based generation with ACE2 as the target”.*

The quantitative estimate of drug-likeness (QED) quantifies the drug-likeness of a molecule using molecular properties as inputs. ^29^ It was calculated by RDKit (2017.09.1) ^30^ and ranges from zero to one, with the closer to one the more favorable.

Synthesize accessibility score (SAscore) measures the difficulty of synthesizing a molecule. ^28^ A predictive model built by Blaschke *et al*. ^13^ was used, where molecular weight was combined with raw score, ^28^ which ranges from one to 10, as features to predict the probability of synthetic accessibility. The model gives a probability score range from zero to one, with the closer to one the better.

Molecular weight and the log of partition coefficient (LogP) were calculated using RDKit. ^30^ Length of the SMILES string was also calculated for the molecules.

### Evaluation settings

The SGPT-RL model was evaluated on a distribution learning benchmark and two tasks for goal-directed generation. The Moses Benchmark was used for distribution evaluation. DRD2 activity and ACE2 affinity were used as the scoring functions in the two goal-directed generations tasks, respectively.

*Benchmarking on distribution learning*

To evaluate on the Moses distribution learning benchmark, the SGPT-RL prior model was trained on Moses training dataset. The model after the final epoch was used to generate 10,000 molecules to evaluate on this benchmark. Five metrics were used for comparison, including validity, uniqueness, novelty, SNN and intDiv. The baseline models from the Moses benchmark were run with default parameters for comparison. MCMG (multi-constraints molecular generation) and MolGPT were also run with default parameters to generate 10,000 molecules for comparison.

*Task 1: goal-directed generation with DRD2 as the target*

In the DRD2 task, we aimed to generate molecules that are active against DRD2. The DRD2 activity predicted by a QSAR model ^12^ was used as the target. The prior model trained from the Moses dataset was used to initiate the agent on this task. The agent was trained for 2,000 steps and the model after the final step was used to sample 10,000 molecules for property distribution analysis.

The Reinvent model ^12^ was used as the baseline in comparison. In this agent, a three-layer GRU was used as the policy model. The default hyper-parameters of Reinvent were used. The prior model was trained for five epochs with a batch size of 128. Adam optimizer was used with a learning rate of 0.001. To train this agent, the same scoring function of the SGPT-RL agent was used for a fair comparison. The Reinvent agent was trained with a batch size of 64, a learning rate of 0.0005, a sigma of 60, and 3,000 steps.

*Task 2: structure-based generation with ACE2 as the target*

In the ACE2 task, we trained the SGPT-RL agent with ACE2 affinity as the desirable property. ACE2 affinity was evaluated by ligand-receptor docking experiments. The 3D structure of the human ACE2 receptor (PDB ID 1R4L) was downloaded from the Protein Data Bank. It was processed with PyMol (2.5.4) ^31^ to remove water molecules and original ligands. An open source of PyMol is available here. The structure was also processed with MGLTools (1.5.7) ^32^ to add polar hydrogen and obtain the docking grid. The pocket where XX5 is located was used to dock with generated molecules. The SMILES strings of generated molecules were used to generate 3D structures of ligands using RDKit (2017.09.1). ^30^ The generated 3D ligand structures were processed with OpenBabel (3.0.0) ^33^ to assign Gasteiger partial charges and convert to pdbqt format. The final docking was performed using AutoDock Vina (1.1.2) ^34^ with eight poses for each ligand. The smallest docking score of the eight poses was used as the docking score of a ligand.

To train the agent, the affinity score was expected to be in a range of zero to one to calculate the augmented log-likelihoods. So the docking score was transformed into a range of zero to one using the reverse sigmoid function as shown in Equation 6, where l , h , and k were constants and set to be -12, -8 and 0.25, respectively.

$$\text{Rsigmoid}\left(x\right)=\frac{1}{1+10^{k*\frac{x-\frac{h+l}{2}}{h-l}}\;}$$ (6)

The Moses pretrained prior model was also used to initiate the agent on this task. The agent was trained for 1,000 steps and 64 molecules were sampled and scored during each step. 10,000 molecules were sampled from the agent model after the final step for property distribution analysis.

The Reinvent model ^12^ was also used as the baseline on this task. The default hyper-parameters of Reinvent were used and the same scoring function of the SGPT-RL agent was used for comparison. This model was trained for 1,000 steps with 64 molecules generated during each step.

### Scaffold analysis

To analyze the scaffold overlaps of the prior models, we clustered the scaffolds of generated molecules and training reference using Butina method in RDKit. ^30^^,^^35^ The molecules from different sources were merged, with invalid and duplicated molecules removed. Murcko Scaffolds were obtained using RDKit and clustered using Morgan fingerprints as inputs. A minimum distance of 0.2 was used during clustering. Venn diagram was used to visualize the number of overlapping clusters and unique clusters. Examples of molecules were visualized using ChemDraw 20.1. ^36^ Some open source alternatives to ChemDraw are available here.

To analyze the average number of rings and the number of explored scaffolds in Figures 3 and 4, RDKit ^30^ was used to obtain the Murcko Scaffold and calculate the number of rings for each generated molecule. The duplicated scaffolds were removed before counting the scaffolds.

**Figure 2.**
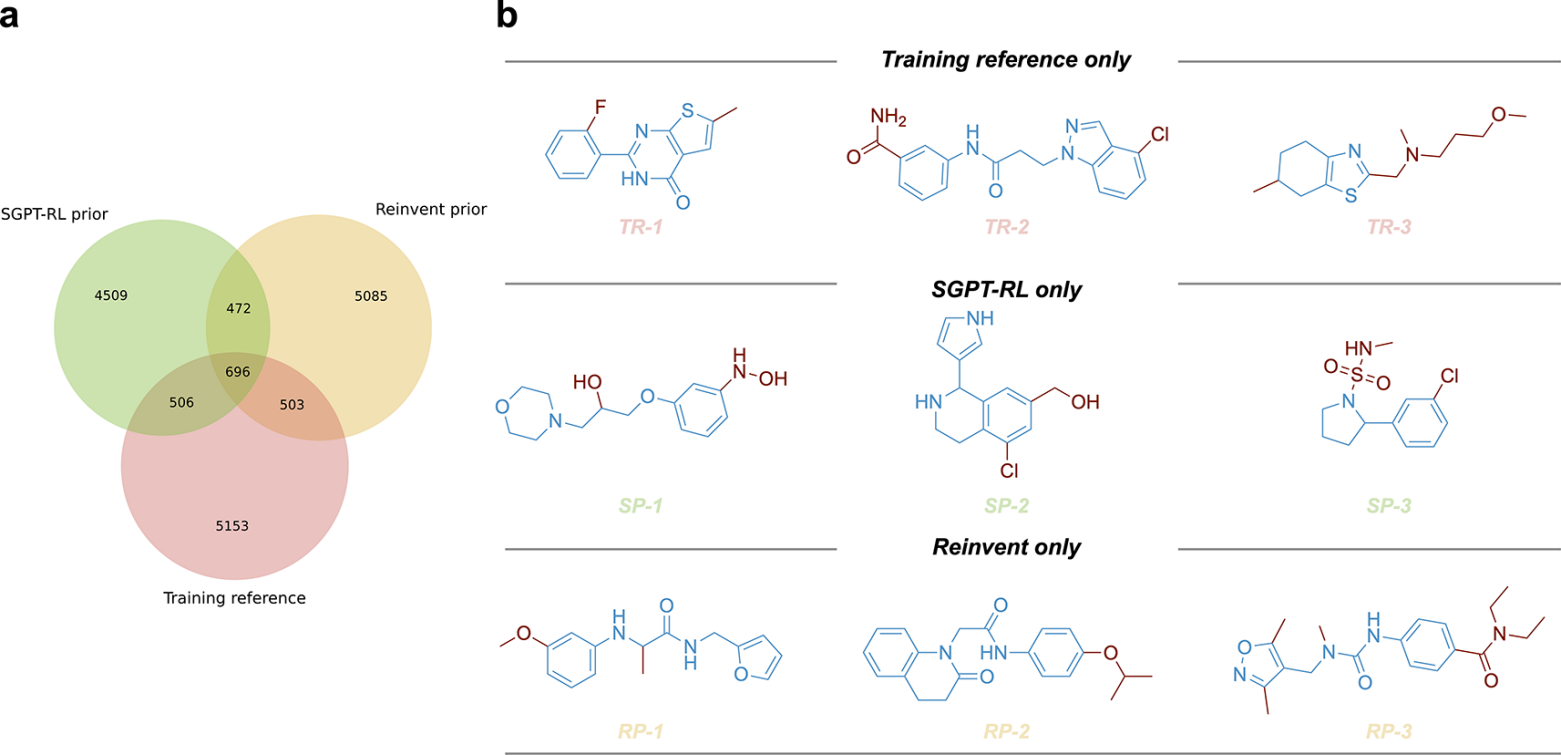
Scaffold overlaps of the prior models. a) The scaffold overlaps between the training reference and molecules generated by the SGPT-RL and Reinvent prior models. Both SGPT-RL and Reinvent were able to generate molecules with novel scaffolds that did not appear in the training reference. b) Representative molecules with unique scaffolds from the three sources. The three rows correspond to training reference only (TR), SGPT-RL prior only (SP), and Reinvent prior only (RP) molecules, respectively.

**Figure 3.**
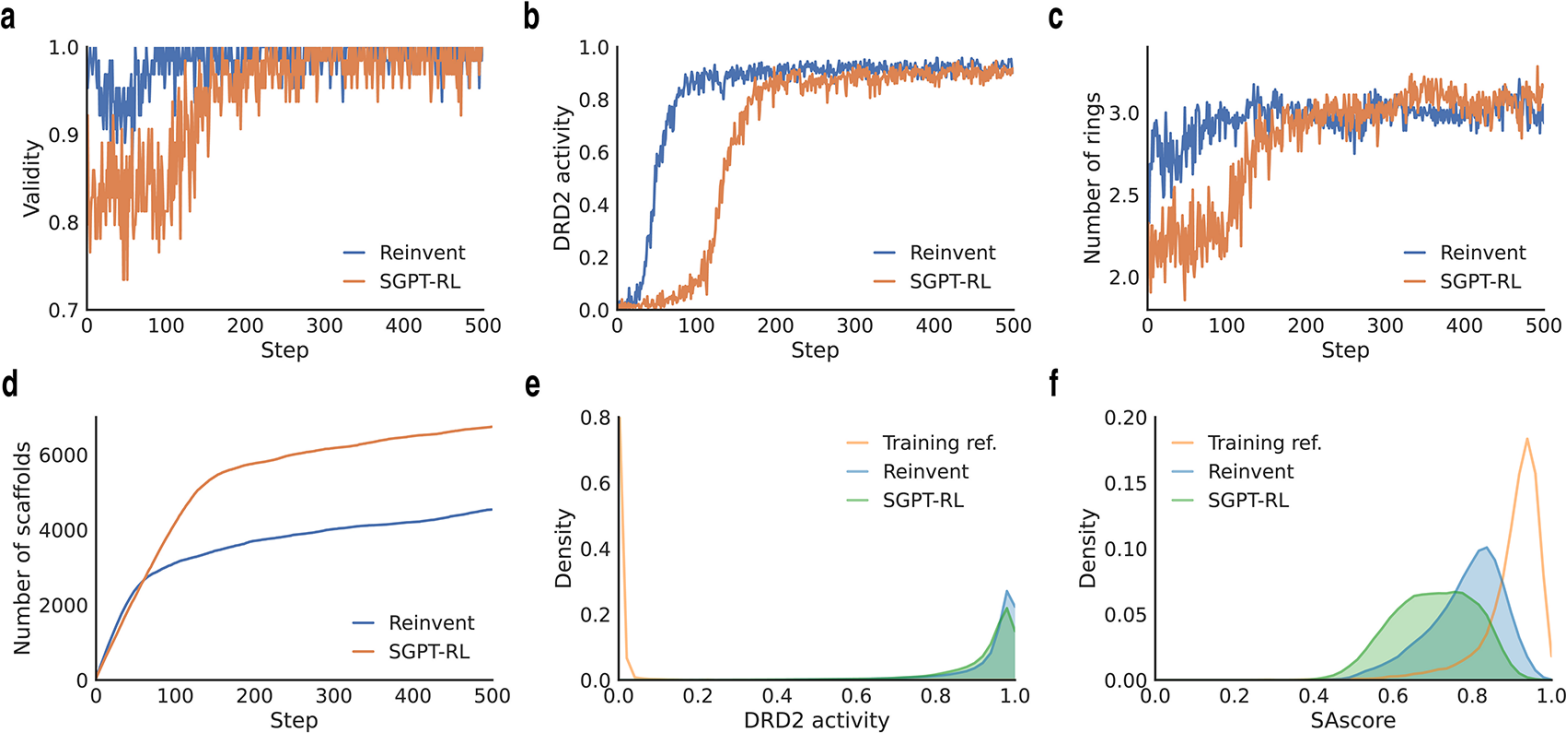
Comparison of SGPT-RL and Reinvent on the DRD2 task. a-b) Improvements of validity and DRD2 activity during the RL process. SGPT-RL was relatively slower in generating molecules with good validity and DRD2 activity than Reinvent. c) Average number of rings in the generated molecules in the RL steps. SGPT-RL gradually increased the number of rings in the generated molecules during the RL process. It generated molecules with fewer rings than Reinvent in the beginning, but with more rings in the end. d) Accumulated number of unique scaffolds in the generated molecules during the RL process. SGPT-RL explored more scaffolds than Reinvent. e) The distribution of predicted DRD2 activities. Both SGPT-RL and Reinvent agents were able to generate molecules with high DRD2 activities. f) The distribution of synthesize accessibility scores (SAscore). 10,000 molecules are sampled from training dataset to be used as the reference (Training ref.).

## Results

### Learning the chemical space with a GPT prior model

The first step of our workflow is to train a prior model to learn the chemical space. To do that, the dataset from the Moses benchmark ^23^ was used to train the prior model. We used Moses dataset because the molecules in this dataset are lead-like molecules and have good chemical properties. A ∼6M GPT model was used as the prior model, details of which are described in Subsection “The prior network”. The Reinvent prior model ^12^ (GRU) was trained on the same dataset for comparison. 10,000 molecules were randomly sampled from the training dataset to be used as the training reference.

A comparison of different models on the Moses distribution learning benchmark ^23^ is shown in Supplementary Table 1 in *Extended data.* ^42^ Five Moses metrics, including validity, uniqueness, similarity to the nearest neighbor (SNN), internal diversity (IntDiv), and novelty, were selected for comparison. From the table, we found that the SGPT-RL prior model achieved a relatively good validity (0.936), uniqueness (0.997), and novelty (0.946). Though the Reinvent prior model achieved a better validity (0.986) and uniqueness (1.000), it obtained a poor novelty (0.783). The other two transformer-based methods, MCMG and MolGPT, also achieved a good novelty (0.983 and 0.931 respectively).

The property distributions of the training reference and molecules sampled from the SGPT-RL and Reinvent prior models were visualized as shown in Supplementary Figure 1 in *Extended data.* ^42^ Six selected properties, including DRD2 activity, ACE2 docking score, QED, synthesize accessibility score (SAscore), length of SMILES strings, and molecular weight were used for comparison. Details on the calculation of these properties are described in Subsection “Evaluated molecular properties”. From this figure, we can see that both prior models learned similar property distributions to the training reference. For molecular weight, the distribution curve of SGPT-RL prior is closer to the training reference than that of the Reinvent prior.

To compare the generative preferences of the SGPT-RL and the Reinvent prior models, we analyzed the scaffolds of the generated molecules. The overlapping scaffolds and unique scaffolds from each source were visualized using a Venn diagram as shown in Figure 2a. From this diagram, we found that both the SGPT-RL and the Reinvent prior models were able to recall scaffolds from the training reference and generate many molecules with novel scaffolds. Several examples of the generated molecules and training samples are shown in Figure 2b.

### Optimizing the scores of a QSAR model through RL

In our experiments, we evaluated SGPT-RL for goal-directed generation with two tasks, a DRD2 task, which used a quantitative structure-activity relationship (QSAR) model ^12^ as the scoring function, and an ACE2 task, which used a docking score calculated from AutoDock Vina ^34^ as the scoring function.

DRD2 is one of the most well-studied drug targets, with many chemicals active against it being reported. ^25^^,^^37^ A QSAR model was proposed for DRD2 activity prediction. ^12^ In this task, the SGPT-RL prior model pretrained on the Moses dataset was used to initiate the agent, and the agent was trained via RL to optimize the generation of molecules towards good DRD2 activities. The Reinvent model was trained with default hyper-parameters for comparison. ^12^ Details on the training of the agents are shown in Subsection “Training the agent”. The hyper-parameter of SGPT-RL was fine-tuned as shown in Supplementary Results in *Extended data.* ^42^ A sigma value of 60 was chosen for this agent.

The learning curves of the agent models on the DRD2 task are shown in Figure 3. From Figures 3a-b, we see that both agents could learn a good validity and DRD2 activity after 200 steps. The Reinvent agent took fewer steps to obtain good DRD2 activity than the SGPT-RL agent. Figures 3c-d show that the SGPT-RL agent gradually increased the number of rings during generation and explored more scaffolds within the first 200 steps. The main difference in scaffold exploration between the two agents is in 100-200 steps. The Reinvent agent was not drastically improving the goal after around 100 steps, while the SGPT-RL agent was continuously learning and improving after that.

The agent models trained after the final step were also evaluated on the Moses benchmark, as shown in Table 1. The Moses metrics of MCMG was also obtained from the original paper for comparison. ^4^ We found that the SGPT-RL agent achieved better validity and novelty, while the Reinvent model obtained a better internal diversity.

**Table 1.** Moses metrics of the agent models on the DRD2 task. SGPT-RL generated molecules with good validity and novelty. SNN, similarity to a nearest neighbor; IntDiv, internal diversity; MCMG, multi-constraints molecular generation.

Model	Validity	Uniqueness	SNN	IntDiv	Novelty
**Reinvent**	0.997	0.880	0.508	**0.709**	0.992
**MCMG**	-	**0.972**	**0.541**	**0.709**	0.992
**SGPT-RL**	**0.998**	0.933	0.515	0.683	**0.995**

The property distributions of the training reference and molecules sampled from the final SGPT-RL and Reinvent agents were also compared in this task, as shown in Figure 3e. ^42^ The properties analyzed include DRD2 activity, QED, SAscore, LogP, length of SMILES strings, and molecular weight. We found that both SGPT-RL and Reinvent could generate molecules with good DRD2 activities after the final steps, whereas the molecules in training reference have poor DRD2 activities. The property distributions of the molecules generated by the SGPT-RL and Reinvent agents are similar. Figure 3f shows that both agents shifted the SAscore distributions to the left, which means generating molecules that are relatively harder to synthesize than the molecules in the training reference.

### Generating molecules to optimize docking scores

In this task, we aimed to generate novel molecules targeting ACE2, a receptor protein which SARS-CoV and SARS-CoV-2 bind to enter a cell. ^38^^,^^39^ Only 56 unique molecules were reported to be active against ACE2 in ExCAPE-DB. ^25^ For such targets where few known active molecules are available, it is not possible to build a reliable QSAR model to predict activity. To find binding molecules against targets like ACE2, structure-based docking methods are widely used to evaluate the affinities. In this study, the ACE2 affinity of a molecule was evaluated as the minimum binding free energy calculated by AutoDock Vina. ^34^ Details on the calculation of ACE2 affinity can be found in Subsection “Evaluated molecular properties”. The pocket, where XX5 is located, in the 3D structure of the human ACE2 receptor (PDB ID 1R4L ^40^) was used to dock with a ligand. The prior model trained on Moses dataset ^23^ was also used to initiate this agent, and the agent was trained for 1,000 steps. The Reinvent model was also trained on this task for a fair comparison.

**Figure 4.**
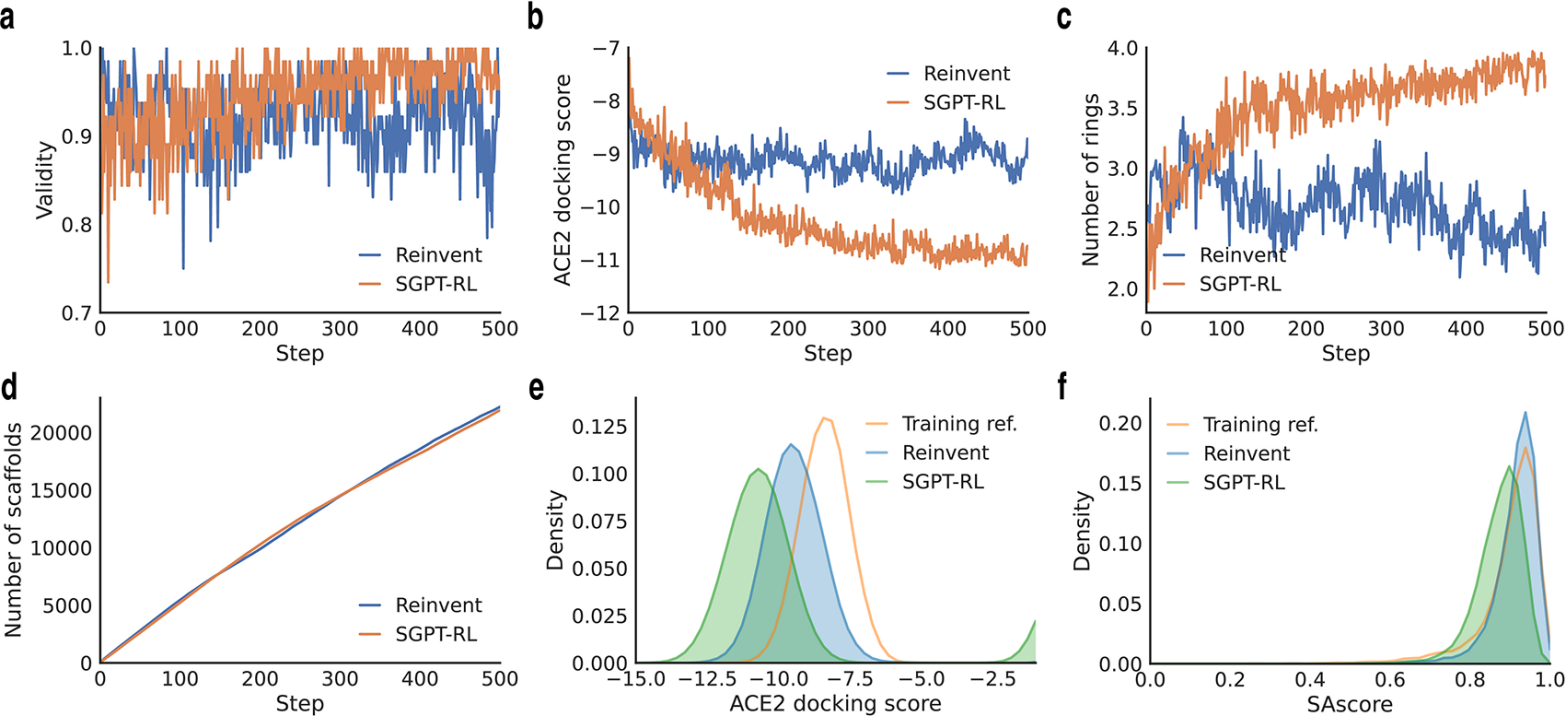
Comparison of SGPT-RL and Reinvent on the ACE2 task. a-b) Improvements of validity and ACE2 docking scores during the RL process. SGPT-RL generated molecules with better validity and ACE2 docking scores than Reinvent after 200 steps. c) Averaged number of rings in the generated molecules in the RL steps. SGPT-RL gradually increased the number of chemical rings of the molecules. The curve difference in c is highly correlated with the curve difference in b (Pearson’s r = 0.87). d) Accumulated number of unique scaffolds in the generated molecules during the RL process. Both SGPT-RL and Reinvent generated new scaffolds with increasing steps. e) The distribution of ACE2 docking scores. SGPT-RL shifted the distribution towards better docking scores. f) The distribution of SAscore.

**Figure 5.**
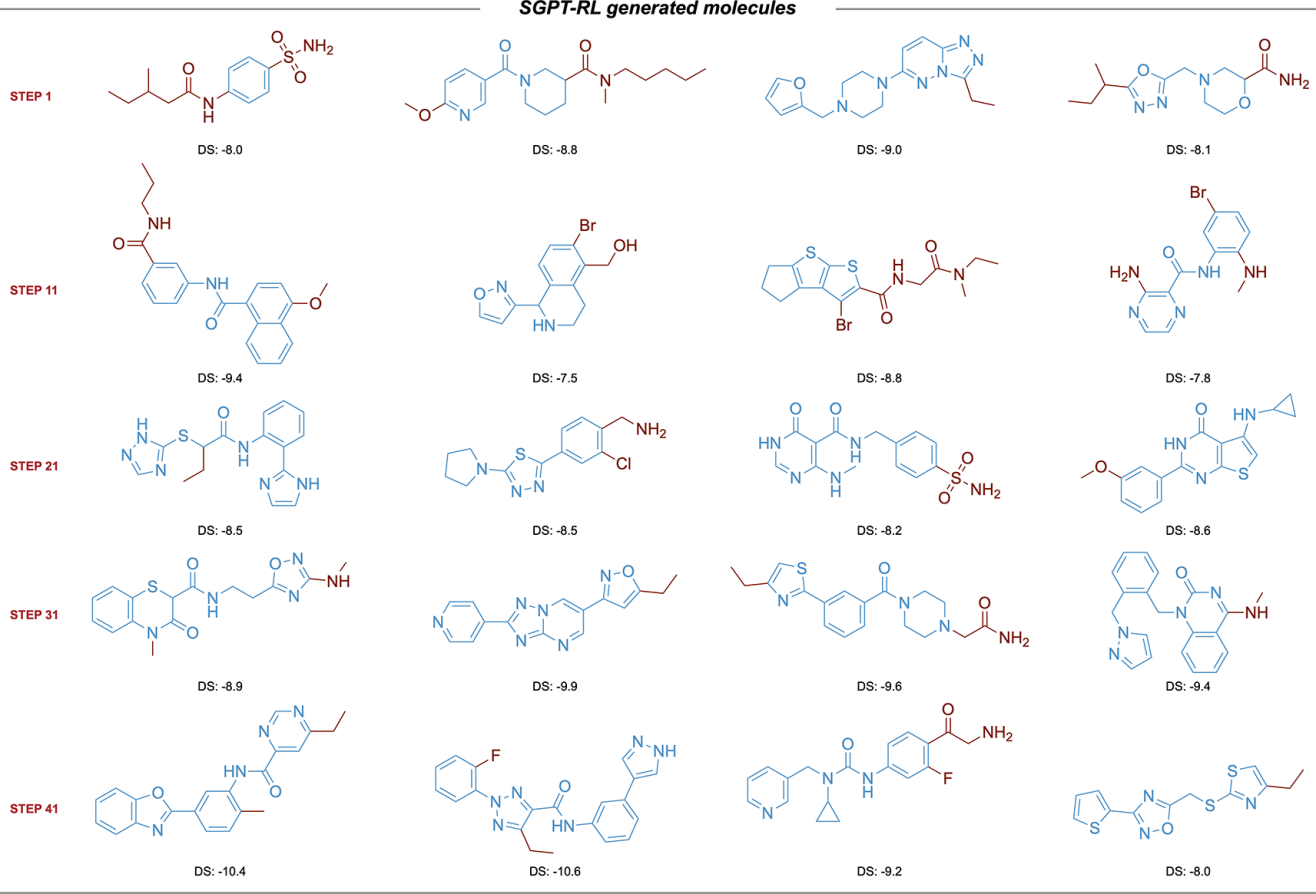
Examples of scaffolds explored by SGPT-RL in the initial steps of the ACE2 task. The SGPT-RL agent generated molecules with few rings in the beginning, and gradually increased the number of rings. DS, docking score.

The learning curves of the agent models are shown in Figure 4. The SGPT-RL agent was able to generate valid molecules with good ACE2 docking scores after 200 steps. Like the DRD2 task, in the ACE2 task the Reinvent model was not efficiently learning after around 100 steps. The docking scores of the generated molecules were not clearly improving after that. Besides, we also observed that SGPT-RL gradually increased the number of rings in the exploration process, as shown in Figure 4c. Examples of molecules generated by SGPT-RL during the initial exploration steps are shown in Figure 5. The SGPT-RL agent generated molecules with few rings in the first step, and gradually increased the number of rings. The Reinvent agent was randomly exploring the molecules, and no clear patterns can be observed, as shown in Supplementary Figure 7 in *Extended data.* ^42^

The final agents were evaluated on the Moses metrics, as shown in Table 2. The SGPT-RL agent achieved good validity (0.990) and novelty (1.000), while Reinvent was better on SNN and internal diversity. The property distributions were plotted for the two agents. Six selected properties, including ACE2 docking score, QED, SAscore, LogP, length of SMILES string, and molecular weight, were analyzed, as shown in Supplementary Figure 8 in *Extended data.* ^42^ Calculations of these properties are described in Subsection “Evaluated molecular properties”. From Figure 4e, ^42^ we see that the SGPT-RL agent was able to generate molecules with good docking scores and clearly shifted the distribution curves to the left. The ACE2 docking scores of SGPT-RL generated molecules were better than the training reference or the Reinvent generated molecules. Supplementary Figure 9 in *Extended data* ^42^ shows some examples of molecules generated by the agents in the last step. SGPT-RL generated molecules are more similar to each other in comparison with Reinvent generated molecules. From these molecules, we can see that SGPT-RL tends to generate with certain preferences, such as a naphthalene structure in one end in this task.

**Table 2.** Moses metrics of the agents on the ACE2 task. SNN, similarity to a nearest neighbor; IntDiv, internal diversity.

Model	Validity	Uniqueness	SNN	IntDiv	Novelty
**Reinvent**	0.875	**0.987**	**0.560**	**0.816**	0.976
**SGPT-RL**	**0.990**	0.986	0.466	0.797	**1.000**

**Figure 6.**
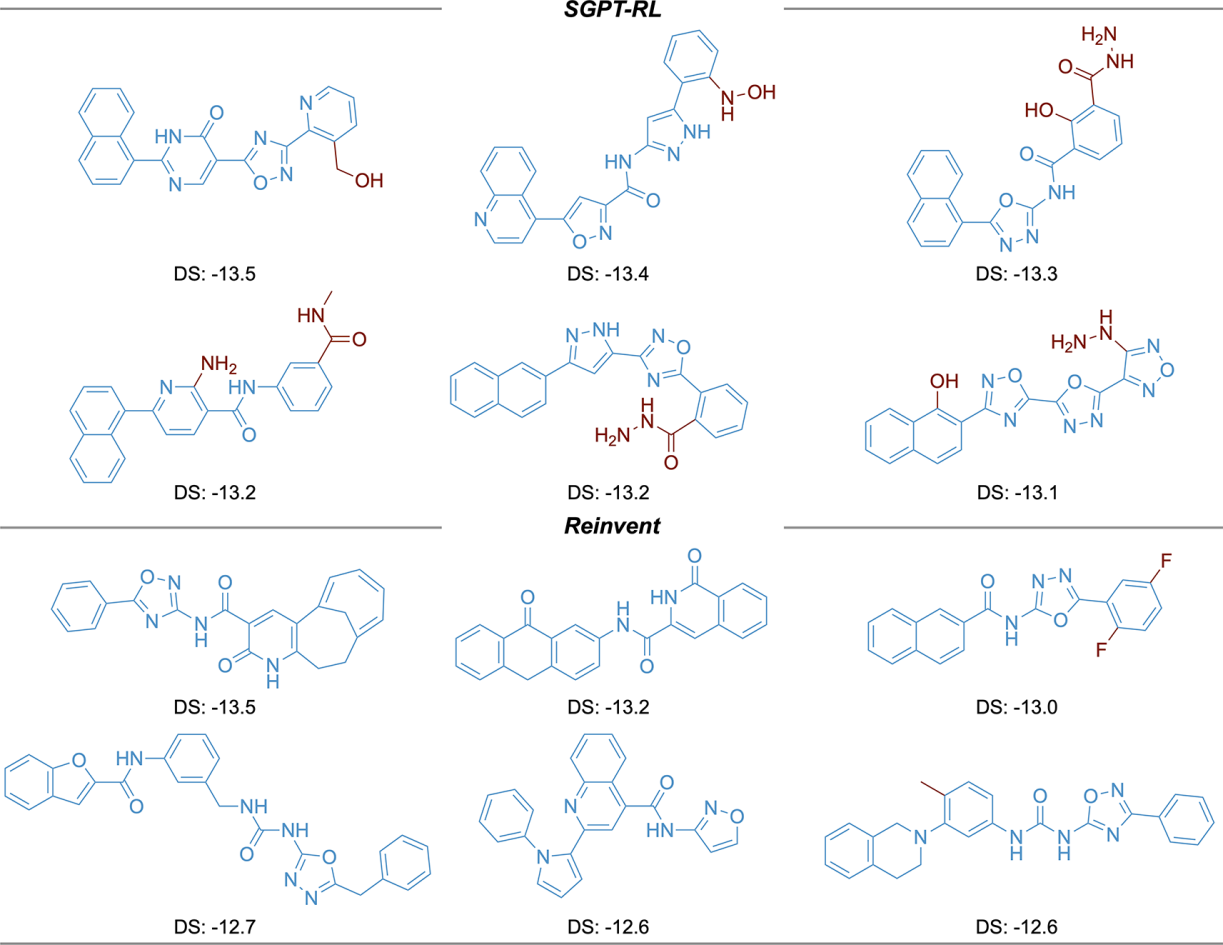
Top scoring molecules generated in the ACE2 task. The SGPT-RL generated molecules are more similar to each other in comparison with the Reinvent generated molecules. DS, docking score.

The top six molecules with the highest docking scores generated by the agents are shown in Figure 6. The SGPT-RL agent was able to generate more molecules with high docking affinities than the Reinvent agent. Besides, five out of the top six molecules generated by SGPT-RL contain a naphthalene structure in one end. Considering the same pattern in the molecules generated by SGPT-RL in the last step, we would guess that the agent had learned such a pattern during the exploration process. However, the top scoring molecules generated by the Reinvent agent have strong randomness and no clear scaffold patterns can be observed.

## Discussion

In this study, we developed a tool named SGPT-RL for de novo molecular generation, which uses a transformer decoder as the policy network of the reinforcement learning (RL) agent. A workstation with two A100 GPUs was used for our experiments. The docking score was used as a scoring function in addition to a QSAR-based scoring function. This enabled us to explore not only a target with many known active molecules but also a new target with few known actives.

We evaluated SGPT-RL on two goal-directed generation tasks, a DRD2 task and an ACE2 task. As many known DRD2 actives are available, it is possible to build a reliable QSAR model to be used as the scoring function in the DRD2 task. However, few known actives were reported for ACE2, so Vina docking scores had to be used as the optimization goal in the ACE2 task. Our experiments showed that both SGPT-RL (which uses GPT as the policy network) and Reinvent (which uses GRU as the policy network) were able to propose molecules with improved scores on the two tasks. However, the SGPT-RL generated molecules showed significantly better scores on the ACE2 task compared to the Reinvent generated ones (p-value: 0.0). As the molecular docking was widely used for the virtual screening process, we believe that the superior performance of SGPT-RL in the ACE2 task would indicate its wide applicability in the practical molecular design procedure.

Besides, we found three generative differences between the SGPT-RL and Reinvent agents during the exploration steps. First, in the experiments, we found that Reinvent was exploring with strong randomness in the two tasks in general, however, SGPT-RL gradually explored the scaffolds during the generation processes. In the initial steps, SGPT-RL generated molecules with few rings and gradually increased the number of rings during exploration; in the late steps, it generated molecules with some conserved scaffold patterns, such as double ring structures in the ACE2 task. Second, we found that Reinvent was not clearly improving the goal after around 100 steps, while SGPT-RL was continuously optimizing the scores even after 400 steps. We believe that this difference is mainly caused by the difference in policy networks: it is not easy for GRU to learn ring patterns, which are represented as distant numbers in SMILES; however, GPT was able to learn long-range dependencies to remember the ring patterns that had improved scores in previous steps. Thirdly, the SGPT-RL agent could generate molecules with more rings than the Reinvent agent in the ACE2 task (shown in Figure 4c). A diverse number of rings indicates a variety of scaffold structures. Considering the importance of appropriate scaffolds in lead identification, ^41^ we believe that including GPT as the policy network in RL agents might be useful to discover lead candidates of novel scaffolds.

While the results of our work are noteworthy, there are two limitations to consider. First, the dataset to train the prior models would be a limit to the generative results. All the prior models were pretrained on the Moses dataset. ^23^ As the Moses dataset was collected from the Zinc database, ^24^ which mainly consists of lead-like molecules, the prior distribution could not represent the entire chemical space. The prior models were used to guide the agents in the two optimization tasks, and the bias in the prior models might contribute to the bias in the agent models. Such bias might be contributive, because it would help to generate molecules with lead-like properties, such as good synthetic accessibility and drug-likeness; however, it might also be undesirable, as it limits the chemical space the agents explored. In tasks which aim to explore out of the space of lead-like molecules, other training data should be utilized to train the prior models. Second, the settings of the docking experiments would also be a limit. We analyzed ACE2 for docking, but docking experiments of additional targets would further confirm the observations in our study.

As molecular docking was widely used for virtual screening, generative models combined with molecular docking provides another solution for the virtual screening process. The superior performance of SGPT-RL on the ACE2 task indicates that it can be applied to this practical molecular design process and propose novel molecules with good target-binding capabilities. Besides, SGPT-RL explored the chemical space with certain scaffold patterns. The patterns learned by SGPT-RL can provide intuitions for chemists to explore, thus aid the molecular design.

## Data Availability

Protein Data Bank: 3D structure of the human ACE2 receptor. Accession number 1R4L; https://www.rcsb.org/structure/1R4L. The dataset to train the prior models was obtained from the Moses benchmark. ^23^ This dataset contains 1.9 million lead-like molecules from the Zinc database, and is available to readers here: https://github.com/molecularsets/moses. The train and test dataset in the Moses benchmark, used here for training and testing, contains 1,584,664 and 176,075 molecules respectively. Moses is licensed under MIT license (redistribution permitted). The 8,036 unique molecules that are known to be active against DRD2 and 56 unique molecules that are active against ACE2 were downloaded from ExCAPE-DB , ^25^ and which are licensed under Creative Commons Attribution 4.0 International License (redistribution permitted). The specific underlying data used in this study been uploaded by the authors to Zenodo (see below). Zenodo: Optimization of binding affinities in chemical space with transformer and deep reinforcement learning -- source data. https://doi.org/10.5281/zenodo.10654313. ^42^ This project contains the following underlying data:
- Data.zip (the Moses dataset, the DRD2 and ACE2 active molecules, the pretrained models, and the source data underlying Figures 3– 4). Data are available under the terms of the Creative Commons Zero “No rights reserved” data waiver (CC0 1.0 Public domain dedication). Zenodo: Optimization of binding affinities in chemical space with transformer and deep reinforcement learning -- source data https://doi.org/10.5281/zenodo.10654313. ^42^ This project contains the following extended data:
- SGPT_SI.pdf (supplementary results, tables, and figures). - Sgpt-rl.png (the workflow of SGPT-RL). Data are available under the terms of the Creative Commons Zero “No rights reserved” data waiver (CC0 1.0 Public domain dedication).

## Software availability

Source code available from: https://github.com/charlesxu90/sgpt

Archived source code at time of publication: https://doi.org/10.5281/zenodo.7612354. ^43^

License: MIT
