# Supplementary material for: Optimization of binding affinities in chemical space with generative pre-trained transformer and deep reinforcement learning
Source: F1000Res. 2024 Feb 20;12:757. Originally published 2023 Jun 28. [Version 2] doi: 10.12688/f1000research.130936.2 (PMC10905145; doi:10.12688/f1000research.130936.2)
Supplement: Peer review history

# Reviewer report: Reviewer response for version 2

**DOI:** 10.5256/f1000research.162639.r248667

**Published:** 2024-02-29

**Authors:** Jianmin Wang

**Affiliations:** 1 Yonsei University, Seodaemun-gu, Seoul, South Korea

No further comment. Thank you for your kind responses.

Is the work clearly and accurately presented and does it cite the current literature?

Yes

If applicable, is the statistical analysis and its interpretation appropriate?

Yes

Are all the source data underlying the results available to ensure full reproducibility?

Yes

Is the study design appropriate and is the work technically sound?

Yes

Are the conclusions drawn adequately supported by the results?

Yes

Are sufficient details of methods and analysis provided to allow replication by others?

Yes

Reviewer Expertise:

drug design, deep learning

I confirm that I have read this submission and believe that I have an appropriate level of expertise to confirm that it is of an acceptable scientific standard.

# Reviewer report: Reviewer response for version 2

**DOI:** 10.5256/f1000research.162639.r248666

**Published:** 2024-02-23

**Authors:** Qifeng Bai

**Affiliations:** 1 Lanzhou University, Lanzhou, Gansu, China

Good work. Please accept it.

Is the work clearly and accurately presented and does it cite the current literature?

Yes

If applicable, is the statistical analysis and its interpretation appropriate?

Yes

Are all the source data underlying the results available to ensure full reproducibility?

Yes

Is the study design appropriate and is the work technically sound?

Partly

Are the conclusions drawn adequately supported by the results?

Yes

Are sufficient details of methods and analysis provided to allow replication by others?

Yes

Reviewer Expertise:

deep learning, binding affinity and drug design,

I confirm that I have read this submission and believe that I have an appropriate level of expertise to confirm that it is of an acceptable scientific standard.

# Reviewer report: Reviewer response for version 2

**DOI:** 10.5256/f1000research.162639.r248664

**Published:** 2024-02-23

**Authors:** Ka-Chun Wong

**Affiliations:** 1 Department of Computer Science, City University of Hong Kong, Kowloon Tong, Kowloon, Hong Kong

The authors have responded.

Is the work clearly and accurately presented and does it cite the current literature?

Yes

If applicable, is the statistical analysis and its interpretation appropriate?

Yes

Are all the source data underlying the results available to ensure full reproducibility?

Yes

Is the study design appropriate and is the work technically sound?

Yes

Are the conclusions drawn adequately supported by the results?

Yes

Are sufficient details of methods and analysis provided to allow replication by others?

Yes

Reviewer Expertise:

Bioinformatics

I confirm that I have read this submission and believe that I have an appropriate level of expertise to confirm that it is of an acceptable scientific standard.

# Reviewer report: Reviewer response for version 1

**DOI:** 10.5256/f1000research.143734.r188009

**Published:** 2023-12-12

**Authors:** Aslıhan Aycan Tanrıverdi

**Affiliations:** 1 Kafkas University, Kars Merkez, Turkey

The authors published the paper entitled "Optimization of binding affinities in chemical space with generative pre-trained transformer and deep reinforcement learning [version 1; peer review: 1 approved with reservations]." The work is very comprehensive. An innovative article worth publishing. I want to congratulate the authors. There's just one point where I'm stuck.

 ***QSAR processing methodology should be given step by step in the methods section.

Is the work clearly and accurately presented and does it cite the current literature?

Yes

If applicable, is the statistical analysis and its interpretation appropriate?

Yes

Are all the source data underlying the results available to ensure full reproducibility?

Partly

Is the study design appropriate and is the work technically sound?

Yes

Are the conclusions drawn adequately supported by the results?

Yes

Are sufficient details of methods and analysis provided to allow replication by others?

Partly

Reviewer Expertise:

- Polymer Synthesis and Characterization- Monomer Synthesis and Ch.- Quantum Chemistry- Molecular Modelling- Molecular Dynamic- Drug Design- Density Functional Theory- Atom in Molecules Analysis- Film Formation- Gel Formation

I confirm that I have read this submission and believe that I have an appropriate level of expertise to confirm that it is of an acceptable scientific standard, however I have significant reservations, as outlined above.

# Reviewer report: Reviewer response for version 1

**DOI:** 10.5256/f1000research.143734.r214522

**Published:** 2023-12-12

**Authors:** Qifeng Bai

**Affiliations:** 1 Lanzhou University, Lanzhou, Gansu, China

In this study, authors use generative pre-trained transformer and deep reinforcement learning to optimize the binding affinities in chemical space. I have some comments as follows:

 1. I have checked the source codes https://github.com/charlesxu90/sgpt. The authors give a nice description for their models. I have an install question. Why do authors repeat to install “openbabel” by command: “sudo apt-get install -y openbabel” even though Conda can install openbabel?

 2. Please check equation 1. There are some kinds of attention formulas. Do authors describe the correct attention formulas for their used pre-trained models?

 3. To make the affinity introduction richer, authors can add more references about binding affinities with deep learning methods such as “Bai, Q, Liu, S, Tian, Y, Xu, T, Banegas-Luna, AJ, Pérez-Sánchez, H, et al. Application advances of deep learning methods for de novo drug design and molecular dynamics simulation. WIREs Comput Mol Sci. 2022; 12:e1581. https://doi.org/10.1002/wcms.1581 “

Is the work clearly and accurately presented and does it cite the current literature?

Yes

If applicable, is the statistical analysis and its interpretation appropriate?

Yes

Are all the source data underlying the results available to ensure full reproducibility?

Yes

Is the study design appropriate and is the work technically sound?

Partly

Are the conclusions drawn adequately supported by the results?

Yes

Are sufficient details of methods and analysis provided to allow replication by others?

Yes

Reviewer Expertise:

deep learning, binding affinity and drug design,

I confirm that I have read this submission and believe that I have an appropriate level of expertise to confirm that it is of an acceptable scientific standard, however I have significant reservations, as outlined above.

# Reviewer report: Reviewer response for version 1

**DOI:** 10.5256/f1000research.143734.r214547

**Published:** 2023-11-01

**Authors:** Jianmin Wang

**Affiliations:** 1 Yonsei University, Seodaemun-gu, Seoul, South Korea

This paper introduces a method called SGPT-RL, which utilizes GPT as the policy network in the Reinvent approach to improve the optimization of binding affinities, such as DRD2 QSAR score and ACE2 docking score. The findings of the study indicate that GPT effectively learns about the chemical space and generates compounds that are both novel and valid, which is consistent with previous research. Furthermore, GPT proves to be proficient in learning ring patterns and successfully explores various scaffolds during the exploration process in both optimization tasks. Particularly in the ACE2 task, SGPT-RL outperforms by achieving superior docking scores and identifying specific patterns, like the presence of double-ring structures.

 The study shows promise overall, with GPT being a robust generative model and drug design being an important area of application for generative AI. The manuscript is well composed, but certain improvements are necessary to address a few issues.

 Major issues:

1. In this study, the authors compared MCMG in the DRD2 task but chose not to include it in the ACE2 task. It seems more logical to compare MCMG in both tasks. However, what might be the rationale behind excluding it from the ACE2 task comparison?
2. The clarity of the presented results is insufficient. In my opinion, Supplementary Figure 8 effectively demonstrates the distributions and should be included in the main content for clear comprehension. Figure 5, on the other hand, would be more suitable to be relocated to the supplementary material.

Minor issues:

1. The manuscript is burdened with too many explanations for common abbreviations, making it a tedious read. For example, the abbreviation "SGPT-RL" is explained repeatedly in each figure, and abbreviations like "RL," "DRD2," and "ACE2" are needlessly reiterated in the captions. It would be more efficient to provide explanations for these abbreviations only when they first appear in the captions, thus avoiding unnecessary repetition.
2. The authors should carefully review the paper to avoid any typos and grammatical errors. Specifically, 'the' should be included before 'Moses benchmark'; 'Similarity to the nearest neighbor (SNN)' in the Subsection 'Evaluation metrics', need to be in lower case; “range in ^1, 10^” should be “range in [1, 10]”.
3. Several spots are not fluent to read. For example, the first sentence in the “Model architecture” Subsection does not fit with the context and should be tuned. “see also Underlying data” doesn’t fit with the context as well.

Is the work clearly and accurately presented and does it cite the current literature?

Yes

If applicable, is the statistical analysis and its interpretation appropriate?

Yes

Are all the source data underlying the results available to ensure full reproducibility?

Yes

Is the study design appropriate and is the work technically sound?

Yes

Are the conclusions drawn adequately supported by the results?

Yes

Are sufficient details of methods and analysis provided to allow replication by others?

Yes

Reviewer Expertise:

drug design, deep learning

I confirm that I have read this submission and believe that I have an appropriate level of expertise to confirm that it is of an acceptable scientific standard, however I have significant reservations, as outlined above.

# Reviewer report: Reviewer response for version 1

**DOI:** 10.5256/f1000research.143734.r188001

**Published:** 2023-11-01

**Authors:** Guohua Wang

**Affiliations:** 1 Key Laboratory of Tree Genetics and Breeding, Northeast Forestry University, Harbin, Heilongjiang, China

In this paper, the authors proposed SGPT-RL, a method that utilizes GPT as the policy network within the Reinvent approach to enhance the optimization of binding affinities, including DRD2 QSAR score and ACE2 docking score. The results of their study demonstrate that GPT effectively learns the chemical space, generating compounds with high novelty and validity, consistent with previous research. Notably, in both optimization tasks, GPT exhibits proficiency in learning ring patterns and successfully explores a wide range of scaffolds during the exploration process. Importantly, SGPT-RL outperforms in the ACE2 task by obtaining superior docking scores and identifying specific patterns, such as the presence of double ring structures.

 Overall, this study is interesting, as GPT is the current hotspot in AI research and de novo drug design is one of the most successful cases in AI for science. The manuscript is also well written and easy to understand. But there are several issues which should be improved.

 Firstly, there are an excessive number of explanations for common abbreviations in this manuscript, which makes it tedious to read. For instance, the abbreviation "SGPT-RL" is repeatedly explained in each of the figures. Similarly, abbreviations like "RL," "DRD2," and "ACE2" are unnecessarily reiterated many times in the captions. I believe it would be more effective to provide explanations for these abbreviations only during their initial occurrence in the captions, thereby avoiding repetitive explanations.

 Secondly, in this study, the authors compared MCMG in the DRD2 task, but not in the ACE2 task. Wouldn't it be more natural to compare it in both tasks? What is the reason for excluding it from the comparison in the ACE2 task?

 Thirdly, while going through the supplementary information, I came across Supplementary Figure 8, which serves as a clear illustration. I believe the author should incorporate it into the main content as it provides a clear explanation of the resulting distributions. Figure 5 should be relocated to the supplementary material instead.

 Furthermore, the author should thoroughly proofread the paper for any typos and formatting errors. For instance, 'the' should be added before 'Moses benchmark'. In the first paragraph of Subsection 'Evaluation metrics', 'Similarity to a nearest neighbor (SNN)' should be corrected to 'similarity to a nearest neighbor (SNN)'.

Is the work clearly and accurately presented and does it cite the current literature?

Yes

If applicable, is the statistical analysis and its interpretation appropriate?

Yes

Are all the source data underlying the results available to ensure full reproducibility?

Yes

Is the study design appropriate and is the work technically sound?

Yes

Are the conclusions drawn adequately supported by the results?

Yes

Are sufficient details of methods and analysis provided to allow replication by others?

Yes

Reviewer Expertise:

Artificial intelligence in bioinformatics

I confirm that I have read this submission and believe that I have an appropriate level of expertise to confirm that it is of an acceptable scientific standard, however I have significant reservations, as outlined above.

# Reviewer report: Reviewer response for version 1

**DOI:** 10.5256/f1000research.143734.r188006

**Published:** 2023-07-20

**Authors:** Ka-Chun Wong

**Affiliations:** 1 Department of Computer Science, City University of Hong Kong, Kowloon Tong, Kowloon, Hong Kong

The authors proposed a method, SGPT-RL, to optimize the SMILES sequences to improve binding affinities through incorporating GPT into a reinforcement learning (RL) framework. The authors trained a GPT model as a prior model to learn the chemical space by pretraining on Moses SMILES, and then trained two RL models, one for DRD2 QSAR scores and the other for ACE2 docking scores, to generate SMILES with good binding affinities. The results show that the GPT prior model learned a good distribution of the chemical space. The RL models were able to generate SMILES sequences with binding affinities. In addition, SGPT-RL generated sequences with better docking scores than Reinvent and able to learn certain patterns during the RL process. There are a few considerations that could be addressed:

 Major issues:

 1. The manuscript includes repetitive explanations of abbreviations, such as SGPT-RL, DRD2, ACE2, and SMILES, throughout the passages and captions. This not only hinders the flow of reading but also makes it tedious to navigate through. To enhance readability, I suggest minimizing the frequency of explanations and providing them only when necessary, particularly upon their initial mention.

 2. GPT is mainly learning distributions, and RL is introducing inductive biases to steer the distributions towards desirable properties. Therefore, I think it is crucial to include a figure that demonstrates how the distribution of these properties evolves. Supplementary Figure 8 addresses this aspect effectively, and I recommend incorporating it into the main context to provide a clear illustration.

 3. The supplementary information should have the same name as the main article, i.e. ‘transformer’ should be ‘generative pre-trained transformer’.

 Minor issues:

 1. In Subsection that explains ”SAscore”, the sentence “which ranges in1, 10” should be “which ranges in [1, 10]”.  

 2. The authors stated that SGPT-RL outperformed Reinvent on the ACE2 task with a significant p-value. However, the p-value is reported as 0.0, which appears as a numerical zero. To accurately represent this score, it would be preferable to present it in 2-digit scientific notation.

Is the work clearly and accurately presented and does it cite the current literature?

Yes

If applicable, is the statistical analysis and its interpretation appropriate?

Yes

Are all the source data underlying the results available to ensure full reproducibility?

Yes

Is the study design appropriate and is the work technically sound?

Yes

Are the conclusions drawn adequately supported by the results?

Yes

Are sufficient details of methods and analysis provided to allow replication by others?

Yes

Reviewer Expertise:

Bioinformatics

I confirm that I have read this submission and believe that I have an appropriate level of expertise to confirm that it is of an acceptable scientific standard, however I have significant reservations, as outlined above.
